# Blood-brain barrier disruption and delivery of irinotecan in a rat model using a clinical transcranial MRI-guided focused ultrasound system

**DOI:** 10.1038/s41598-020-65617-6

**Published:** 2020-05-29

**Authors:** Nathan McDannold, Yongzhi Zhang, Jeffrey G. Supko, Chanikarn Power, Tao Sun, Natalia Vykhodtseva, Alexandra J. Golby, David A. Reardon

**Affiliations:** 1 Department of Radiology, Brigham and Women’s Hospital, Harvard Medical School, Boston, MA USA; 2 Department of Medicine, Massachusetts General Hospital, Harvard Medical School, Boston, MA USA; 3 Department of Neurosurgery, Brigham and Women’s Hospital, Harvard Medical School, Boston, MA USA; 4 Department of Medical Oncology, Dana-Farber Cancer Institute, Boston, MA USA; 5 Department of Medicine, Brigham and Women’s Hospital, Harvard Medical School, Boston, MA USA

**Keywords:** CNS cancer, Translational research, CNS cancer, Blood-brain barrier

## Abstract

We investigated controlled blood-brain barrier (BBB) disruption using a low-frequency clinical transcranial MRI-guided focused ultrasound (TcMRgFUS) device and evaluated enhanced delivery of irinotecan chemotherapy to the brain and a rat glioma model. Animals received three weekly sessions of FUS, FUS and 10 mg/kg irinotecan, or irinotecan alone. In each session, four volumetric sonications targeted 36 locations in one hemisphere. With feedback control based on recordings of acoustic emissions, 98% of the sonication targets (1045/1071) reached a pre-defined level of acoustic emission, while the probability of wideband emission (a signature for inertial cavitation) was than 1%. BBB disruption, evaluated by mapping the R1 relaxation rate after administration of an MRI contrast agent, was significantly higher in the sonicated hemisphere (P < 0.01). Histological evaluation found minimal tissue effects. Irinotecan concentrations in the brain were significantly higher (P < 0.001) with BBB disruption, but SN-38 was only detected in <50% of the samples and only with an excessive irinotecan dose. Irinotecan with BBB disruption did not impede tumor growth or increase survival. Overall these results demonstrate safe and controlled BBB disruption with a low-frequency clinical TcMRgFUS device. While irinotecan delivery to the brain was not neurotoxic, it did not improve outcomes in the F98 glioma model.

## Introduction

Disrupting the blood-brain barrier (BBB) with focused ultrasound (FUS) and circulating microbubbles has been investigated to noninvasively and locally enhance drug delivery to the central nervous system^1^. The method has been studied in numerous studies in animals^2–5^ and has begun to be tested in humans to enhance chemotherapy delivery to brain tumors^6,7^ and to reduce amyloid in Alzheimer’s disease patients^8^. While the exact mechanism of action is not fully understood, the disruption appears to arise via the mechanical interactions between the ultrasound field, the circulating microbubbles, and the vasculature that results in an opening of the BBB that lasts for several hours^9^. FUS-induced BBB disruption has been shown to enable delivery of drugs, antibodies, nanoparticles, gene therapies, and even cells^10^.

Brain tumors do not have a fully-intact BBB. However, preclinical studies have shown that FUS can increase the permeability of the partially-intact blood-tumor barrier (BTB) and enhance the delivery of chemotherapy to CNS tumors^11–15^. Perhaps more important is the prospect of getting drugs to surrounding areas where tumor cells are infiltrating into healthy brain where the BBB is intact. This infiltration, which often cannot be fully resected, leads to recurrence and is one of the main challenges in treating patients with brain tumors.

BBB disruption is now being tested clinically^7,8,16^. The ultrasound frequency used in clinical TcMRgFUS systems are lower than those used in most preclinical studies in rodents. A low frequency is used clinically to facilitate transmission through the thicker human skull. Reducing the frequency also increases the volume of the focal region volume and extends the range that can be used for electronic beam steering with phased array transducers. However, a larger focal region can be challenging in the brain in rodents. Reflections and standing waves within the skull cavity can lead to unpredictable hotspots in the resulting acoustic field^17^. If the geometric gain of the transducer is not high enough, the size of the focal region will exceed the dimensions of the brain, leading to high acoustic intensities near the skull due to internal reflections. Due to reflections, the maximum pressure amplitude appears adjacent to the skull. To achieve BBB disruption at center depths, the pressure next to the skull can exceed damage thresholds.

It is important to perform preclinical studies with the same device that will be used clinically. Using large animal models with low-frequency systems can avoid these issues^3,4,18,19^, but such experiments are labor-intensive and expensive. Furthermore, brain tumors and other models for other CNS disorders in large animals are limited. Regulatory agencies may also require preclinical data be obtained with the same equipment planned for human use.

Here we tested the ability of a clinical TcMRgFUS system to reliably disrupt the BBB in rats and enhance delivery of the chemotherapy agent irinotecan (IN) without clinically-significant neurotoxicity. We used a previously-described^20^ closed-loop feedback system based on recordings of acoustic emissions provided real-time feedback control during electronic beam steering. We show that the large geometric gain achieved with a hemispherical transducer results in FUS-induced BBB disruption that can be reliably and repeatedly used in rats. Further, we measured FUS-mediated delivery of IN to the healthy brain and a rat glioma model. We show that we can safely increase delivery of the agent, but that levels of the active metabolite are low, and the enhanced delivery was insufficient to improve outcomes in a rat glioma model.

## Results

### Controller and acoustic emissions

**Figure 1:**
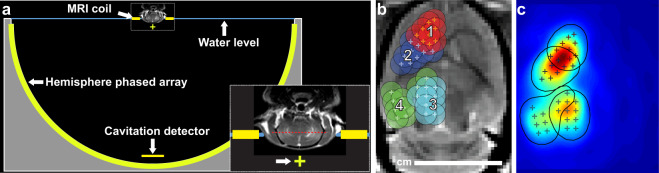
Experimental setup. (**a**) Diagram showing the 30 cm diameter hemisphere phased array with a coronal MRI of the rat head superimposed. The top of the head was partially submerged. A passive cavitation detector was placed near the bottom of the array. The geometric focus (green+) was approximately 1 cm below the target axial plane in the rat brain (red dotted line in inset). (**b**) Locations of the 36 individual sonication targets in each animal. These spots were targeted over four volumetric sonications, in which the phased array electronically steered the focus to nine targets. The circles indicate a 2.5 mm diameter region around each target that determined the extent of where BBB disruption was measured at the end of the experiments. (**c**) Map of the relative acoustic energy density delivered to the brain, calculated by summing intensity maps simulated for each sonication target.

**Figure 2:**
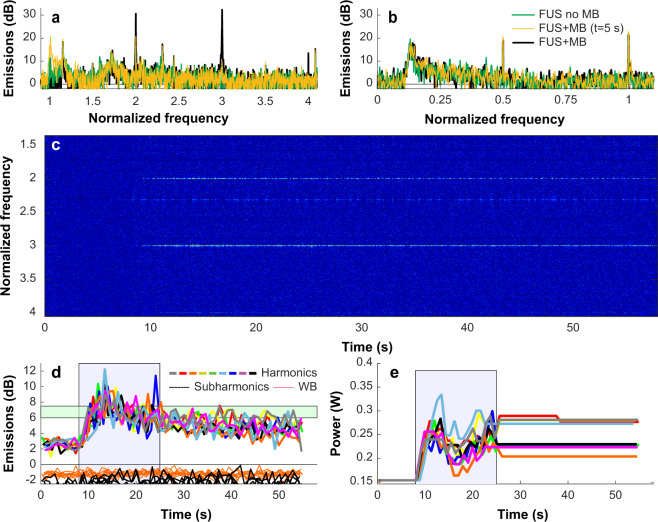
Example acoustic emission obtained during a volumetric sonication with closed-loop feedback control. (**a**,**b**) Example spectra obtained during individual bursts during sonications with and without microbubbles (MB) for the two passive cavitation detectors. Spectra obtained early in the sonication before the microbubbles arrived the brain were similar to those obtained in prior sonications performed without microbubbles. With microbubbles, large and obvious enhancement at the second and third harmonics was observed (**a**), while subharmonic emission was unchanged (**b**). Data are shown in dB relative to the noise floor obtained before the sonication started. (**c**) Spectrogram obtained during this sonication. Here, the data is shown in dB relative to the mean spectra of the first 8 seconds during the sonication with microbubbles. Normalizing the data to spectra obtained before the microbubbles arrived in the brain removed everything except for the harmonic emissions, highlighting the unique signals produced by the microbubbles in the ultrasound field. (**d**) Magnitude of the harmonic, subharmonic, and wideband (WB) emissions plotted as a function of time during the 9 locations targeted in a volumetric sonication. The magnitude of the harmonic emissions were similar for the 9 locations, and all achieved the controller goal of 6–7.5 dB above the noise floor (blue region). Some overshoot occurred. Only small changes were observed in the subharmonic emissions, and no wideband emissions were detected. (**e**) Acoustic power level as a function of time for the 9 locations. During the control period (green region), the power at each location was modified based on the magnitude of the harmonic emissions. After this time, the power level was fixed to the average value of all the bursts that were within the controller goal. In cases where subharmonic or wideband emissions above the noise floor were detected, the power level was reduced by 40% and fixed for the remainder of the sonication.

The cavitation detector was mounted at the bottom of the TcMRgFUS system (Fig. 1a). Example spectra and plots of acoustic emissions in different frequency bands as a function of time are shown in Fig. 2. These data were recorded during one volumetric sonication that focused at nine locations in succession. In each animal, four volumetric sonications were applied to disrupt the entire cerebrum in one hemisphere in a central axial plane (Fig. 1b). Overall, the ten animals in the FUS + IN and FUS-only groups each received three sessions of BBB disruption. Before any microbubbles were administered to the animal, 30 s sonications were performed at the four locations without microbubbles. They were then repeated with the microbubble injection occurring at the start of the sonication.

For the first 8 seconds of the second sonication, the power level was fixed. Spectra obtained here were similar to those acquired in the first sonication without microbubbles (Fig. 2a,b). Between 8–25 s, the power level was increased until the magnitude at the second and third harmonic was between 6–7.5 dB above the noise floor (Fig. 2d,e). In almost every case, obvious and clear enhancement of these harmonic emissions was observed. The emissions reached the controller goals of 6–7.5 dB above the noise floor in 98% of the sonicated locations. They decreased monotonically starting near the end of the control period.

Increased subharmonic or wideband emissions were observed after injection of microbubbles in less than 5% of the sonicated locations (and less than 0.1% of all bursts), and strong harmonic emissions was rarely observed during the sonications without microbubbles. The four locations where the volumetric sonications were applied had similar success rates in achieving the goals of the controller. However, the fourth location had a higher probability for wideband emissions (5.6%, compared to 0.6–2.2% for locations 1–3), and the first location reached the maximum allowed power 23% of the time, compared to only 0.2–12% for locations 1–3. A summary of findings from the acoustic emissions recordings is shown in Table 1.

**Table 1:** Controller performance over 30 consecutive sessions of FUS-induced BBB disruption.

		All		Loc. 1		Loc. 2		Loc. 3		Loc. 4	
Harmonic emission achieved controller goal (locations)	No MB	3.8%	(40/1044)	1.2%	(4/342)	1.2%	(4/342)	8.5%	(29/342)	2.9%	(10/342)
	MB	98%	(1045/1071)	95%	(342/360)	99%	(356/360)	99%	(357/360)	99%	(339/342)
Subharmonic emission (bursts)	No MB	0.40%	(96/26208)	0.2%	(15/8609)	0.4%	(32/8459)	0.5%	(42/8463)	0.3%	(27/8457)
	MB	0.08%	(46/54879)	0.07%	(12/18478)	0.07%	(13/18481)	0.1%	(20/18478)	0.09%	(16/17458)
Subharmonic emission (locations)	No MB	8.4%	(88/1044)	4.4%	(15/342)	8.2%	(28/342)	12.0%	(41/342)	6.7%	(23/342)
	MB	4.2%	(45/1071)	3.1%	(11/360)	3.3%	(12/360)	5.6%	(20/360)	4.7%	(16/342)
Wideband emission (bursts)	No MB	0.02%	(6/26208)	0.05%	(4/8609)	0.0%	(0/8459)	0.02%	(2/8463)	0.0%	(0/8457)
	MB	0.06%	(30/54879)	0.04%	(7/18478)	0.01%	(2/18481)	0.04%	(8/18478)	0.10%	(20/17458)
Wideband emission (locations)	No MB	0.6%	(6/1044)	1.2%	(4/342)	0.0%	(0/342)	0.6%	(2/342)	0.0%	(0/342)
	MB	2.7%	(29/1071)	1.9%	(7/360)	0.6%	(2/360)	2.2%	(8/360)	5.6%	(19/342)
Bursts at maximum power	MB	12%	(6657/54879)	23%	(4208/18478)	12%	(2145/18481)	0.2%	(36/18478)	4.3%	(751/17458)

(MB: microbubbles).

**Figure 3:**
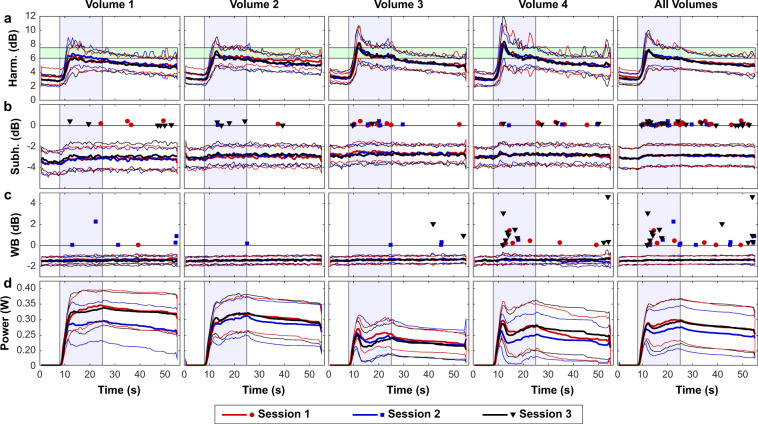
Mean acoustic emissions and acoustic power vs. time for all animals for the four volumetric sonications. (**b**) Harmonic emissions were the basis to control the power level at each target to achieve a pre-determined level between 6–7.5 dB above the noise floor. The controller began 8 seconds after the start of the sonication to allow time for the microbubbles to reach the brain; it was not allowed to increase the power after 25 seconds to avoid increasing the power as the microbubbles were cleared from circulation. Overall, the levels achieved were similar for the four locations targeted, for the three sessions, and for the different animals in the drug distribution study. Some overshoot was evident, particularly in locations 3 and 4. (**c**) Subharmonic emissions, plotted here in dB relative to the threshold used to trigger a reduction in acoustic power, were not reliably detected. The triggers that occurred are noted by the filled symbols and generally were only occurred at an amplitude slightly above this threshold. (**d**) Broadband emission, again plotted here in dB relative to the threshold that triggered a reduction in power, rarely occurred. When it did, its magnitude varied, and it was more likely in location four during the control period. (**e**) The acoustic power varied substantially from sonication to sonication and among the different sessions. However, its trajectory over time was similar for the different sessions. Location 3 consistently required a lower power level. (Thick lines: mean values; thin lines/shaded areas: ±1 standard deviation).

Figure 3 plots the average acoustic emissions and acoustic power as a function of time for all the sonications for the four volumetric targets. The different curves show the average emissions obtained during each of the three weekly sessions for each target (mean ± S.D.). The ability of the controller to drive the microbubbles to a pre-determined level of harmonic emission is evident, as well as the variability in the power required to achieve this level of emissions. Note, for example, the lower power level needed for location 3 that occurred repeatedly in each of the sessions. There was some overshoot in the harmonic emissions in many of the targets, and the increased probability for wideband emissions at location 3 is evident. The corresponding plot for sonication without microbubbles is shown in Supplemental Fig. 1. The probability of detecting subharmonic emissions without microbubbles was higher than during sonication with microbubbles, reflecting the higher acoustic power level or perhaps the presence of small gas bubbles on the skin that dissolved over time or were disrupted by the sonications.

### MRI

**Figure 4:**
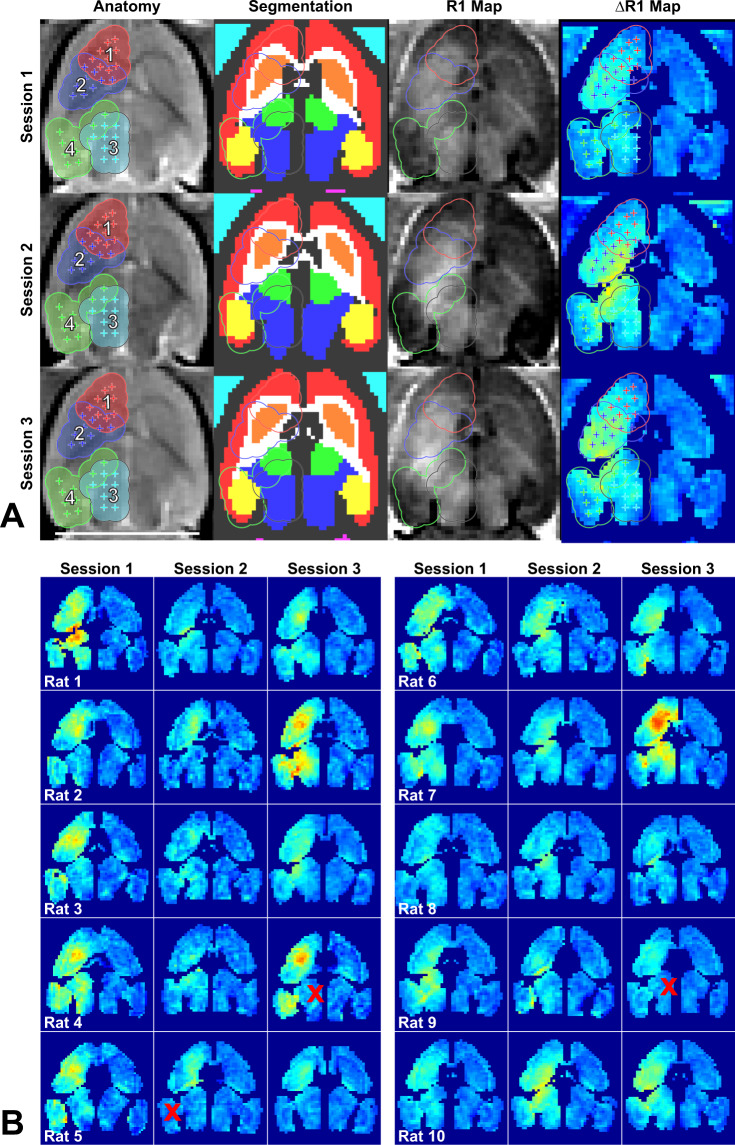
R1 mapping to visualize Gadavist delivery across the BBB over three sessions in 10 rats. (**a**) Anatomic images, segmentations of different brain structures, R1 maps and ΔR1 maps for one rat over three weekly sessions. The 36 sonication targets are indicated on the anatomy images. The contours indicate a 1.25 mm radius around each target for the four volumetric sonications. These contours, along with segmentations of different structures, were used to create ΔR1 maps. The ΔR1 maps show differences in R1 between the sonicated and control hemisphere. (In the segmentation: red = cortex, orange = striatum, white = white matter, green = thalamus, blue = pons, yellow = hippocampus, cyan = non-sonicated muscle) (**b**) ΔR1 maps for 30 consecutive sessions in ten rats. While there was considerable variability, in almost every tissue structure within the sonicated volumes the mean R1 was significantly (P < 0.05) higher when comparing the two hemispheres. The difference was not significant in the thalamus in two sessions and in the hippocampus in one session (indicated by red “x” symbols).

Example MRI obtained during the experiments, along with segmentation of different brain structures and calculated R1 maps are shown in Fig. 4a. Registration of the MRI to an atlas during the experiments ensured that the sonication targets were similarly placed in the brain for the three sessions. Delivery of Gadavist across the BBB was evident in the R1 maps in nearly every sonicated volume and session (Fig. 4b). A significant difference in R1 (P < 0.05) between hemispheres was evident in every tissue structure included in the sonications, except in two cases in the thalamus and one in the hippocampus (red ‘x’ in Fig. 4b).

**Figure 5:**
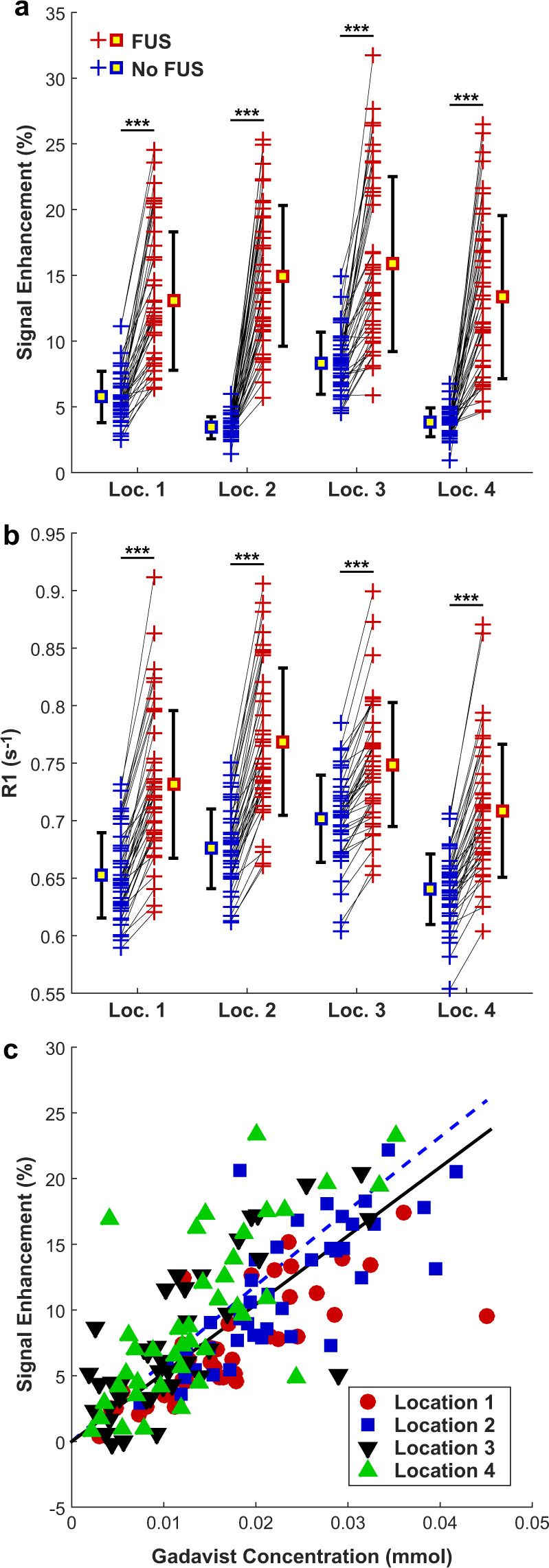
(**a**) Mean changes in MRI signal intensity in T1-weighted imaging after Gadavist injection for the four sonicated locations and the corresponding non-sonicated targets in the contralateral hemisphere for all rats and FUS sessions. The signal change was greater in the sonicated hemisphere in all but one of the sonicated locations. (**b**) Mean R1 relaxation time measured in these four locations. In every case, R1 was larger in the sonicated hemisphere. (**c**) Plot showing the change in signal enhancement in T1-weighted imaging as a function of the Gadavist concentration, which was estimated from the difference in R1 between the sonicated and non-sonicated hemispheres and the relaxivity of the contrast agent (4.44 s^−1^· mM^−1^). The solid line shows the results of a linear regression through zero (R^2^: 0.53). The dotted line shows the expected signal intensity change in this range of Gadavist concentrations, based on the equation for the signal intensity of a spin-echo sequence, the sequence parameters, and the R1 and R2 values of brain tissue. (***P < 0.001).

There was considerable variability in both contrast enhancement and in the calculated R1 values among the different sessions. However, the mean signal enhancement and R1 increases were all positive after Gadavist delivery for the four sonicated regions (Fig. 5a,b). A good correlation (R^2^: 0.52) was observed between the signal enhancement in the T1-weighted imaging and the Gadavist concentrations obtained via the R1 measurements, as shown in Fig. 5c. This plot used the differences in R1 estimates between the sonicated and control hemispheres and the relaxivity of Gadavist (4.44 mmol/s^−1^) to estimate the concentration of the contrast agent in the brain^21^.

**Figure 6:**
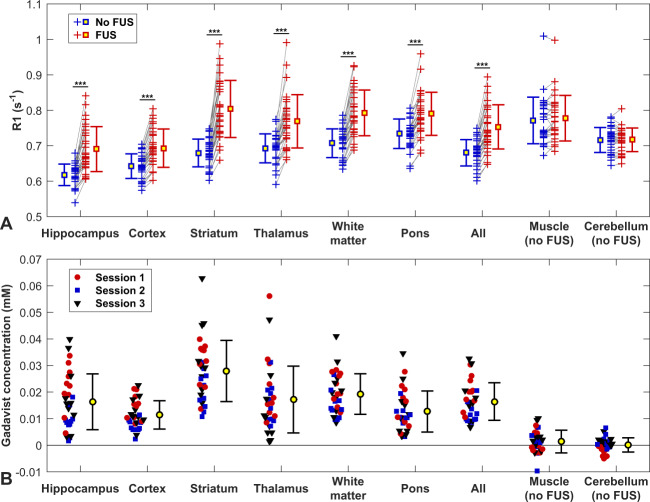
(**a**) Changes in R1 relaxation rate due to the delivery of Gadavist across the BBB for different structures for all animals in this study. Each + indicates the mean signal measured in that tissue structure in the sonicated hemisphere and in corresponding locations in the contralateral, non-sonicated hemisphere. In every individual sonication, R1 was higher in the sonicated side, and overall the difference between the sonicated and control hemispheres was significant for each structure (***P < 0.001). Regions in neighboring muscle and in the cerebellum were not sonicated, and the differences in R1 were not significant. (**b**) Difference in Gadavist concentration between the sonicated and control hemispheres, estimated using the R1 measurements and the relaxivity of the contrast agent (4.44 s^−1^· mM^−1^).

Figure 6 shows the differences in R1 values obtained in different tissue structures in the sonicated vs. non-sonicated hemispheres. Significant changes were observed in the sonicated regions but not in the cerebellum or in muscle, which did not receive FUS. The difference in R1 was significantly higher in the striatum than in the other sonicated targets (P < 0.05).

**Figure 7:**
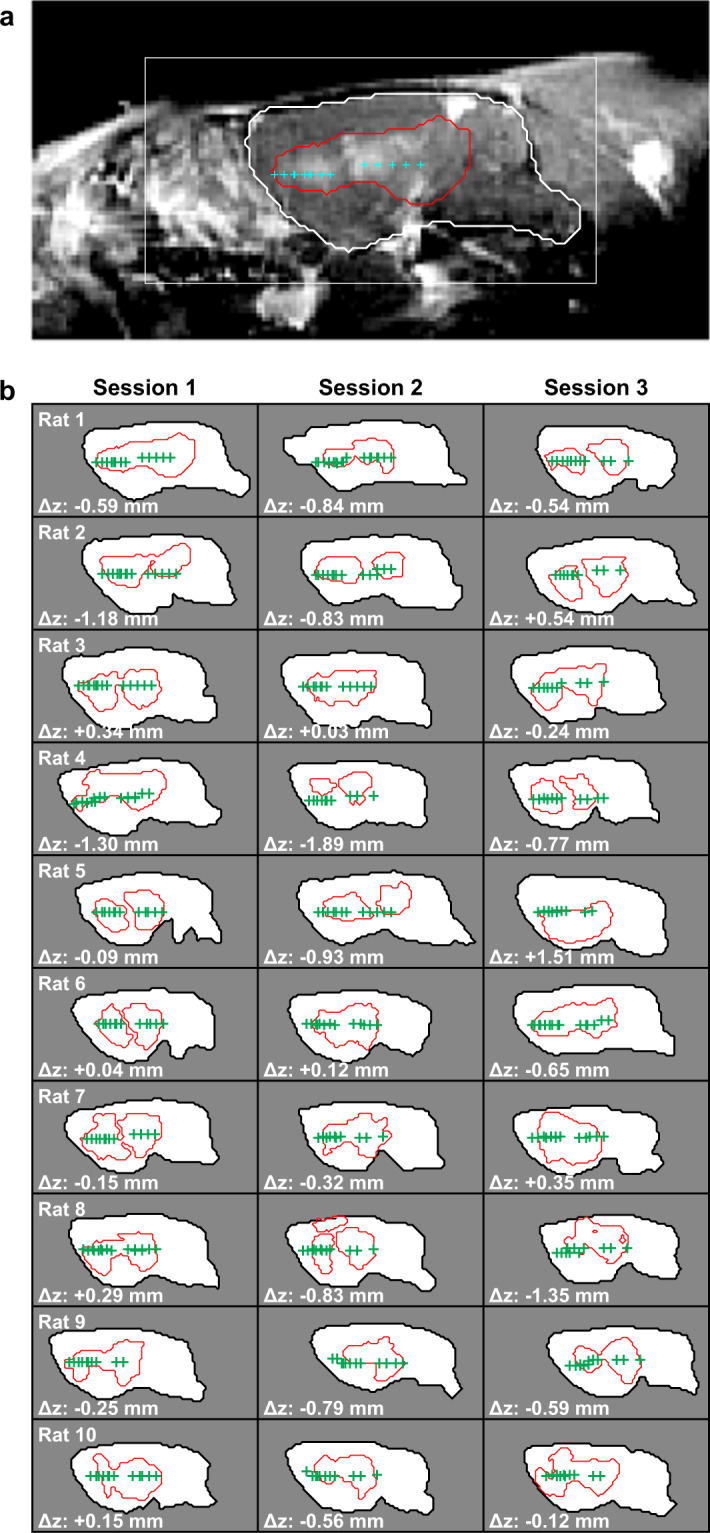
(**a**) Sagittal contrast-enhanced T1-weighted MRI showing BBB disruption, evident in the area outlined in red. The targets that were within ±1 mm of this image plane are shown, and the brain is segmented with a white contour. (**b**) Segmentations of the brain and the area with evident BBB disruption for 30 consecutive sessions. The targets are indicated. In most cases, the extent of the BBB disruption was contained within the brain. In a few cases, the disruption reached the brain surface proximal to the transducer. The distance between the average depth of the segmented BBB disruption and the target plane was less than one millimeter in 25/30 sessions.

Comparison of T2*-weighted MRI obtained before and after sonication revealed no obvious hypointensities that would indicate vascular damage and petechiae. However, there were suspicious faint changes in location 1 after four sessions. Sagittal imaging revealed that the disruption was contained within the brain (Fig. 7) in most animals. In some of the more lateral locations, the disruption reached the brain surface proximal to the transducer. The length of the disruption along the direction of the FUS beam was 3.5 ± 0.7 mm. The mean absolute difference between the center of the disrupted region and the planned depth was 0.6 ± 0.5 mm. This targeting error in this direction ranged from -1.9 to 1.5 mm and was less than one millimeter in 25/30 sessions.

**Figure 8:**
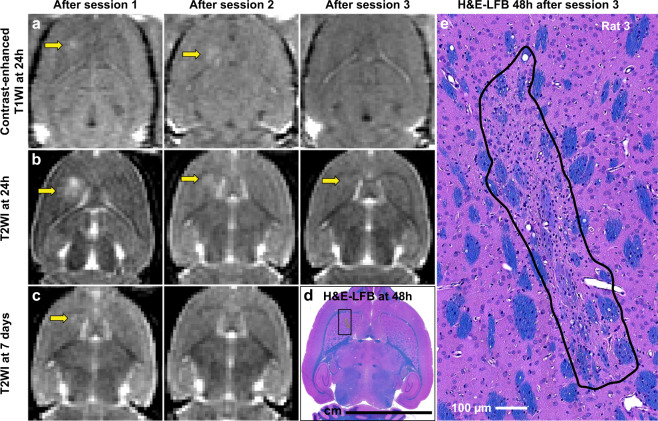
(**a**–**c**) Axial contrast-enhanced T1-weighted MRI and T2-weighted MRI showing BBB disruption and edema, respectively, at 24 hours and seven days after sonication. In this animal, BBB disruption was evident in the striatum (sonicated volume #1) 24 hours after the first two sessions, but not after the third. Edema was evident after the first session. The location with the most severe edema after the first session persisted and was evident in histology (**d**,**e**) as a small pale-stained scar (dimensions: 1.2 × 0.27 mm).

T2-weighted imaging obtained 24 hours after sonication revealed edema in location 1 in 15/30 sessions. In three animals, this edema was not resolved in imaging obtained before the next FUS session. BBB disruption was evident in location 1 at 24 hours in 12/30 sessions. In four of these cases, the BBB disruption was evident without corresponding edema. No edema or BBB disruption was evident in locations 2–4. Example MRI for an animal where BBB disruption and edema was observed at 24 h is shown in Fig. 8a–c. In this animal, a tiny hyperintense area in T2-weighted imaging persisted for the next two weeks. A small pale-stained area was evident in location 1 in histology (Fig. 8d,e).

### Histology

**Figure 9:**
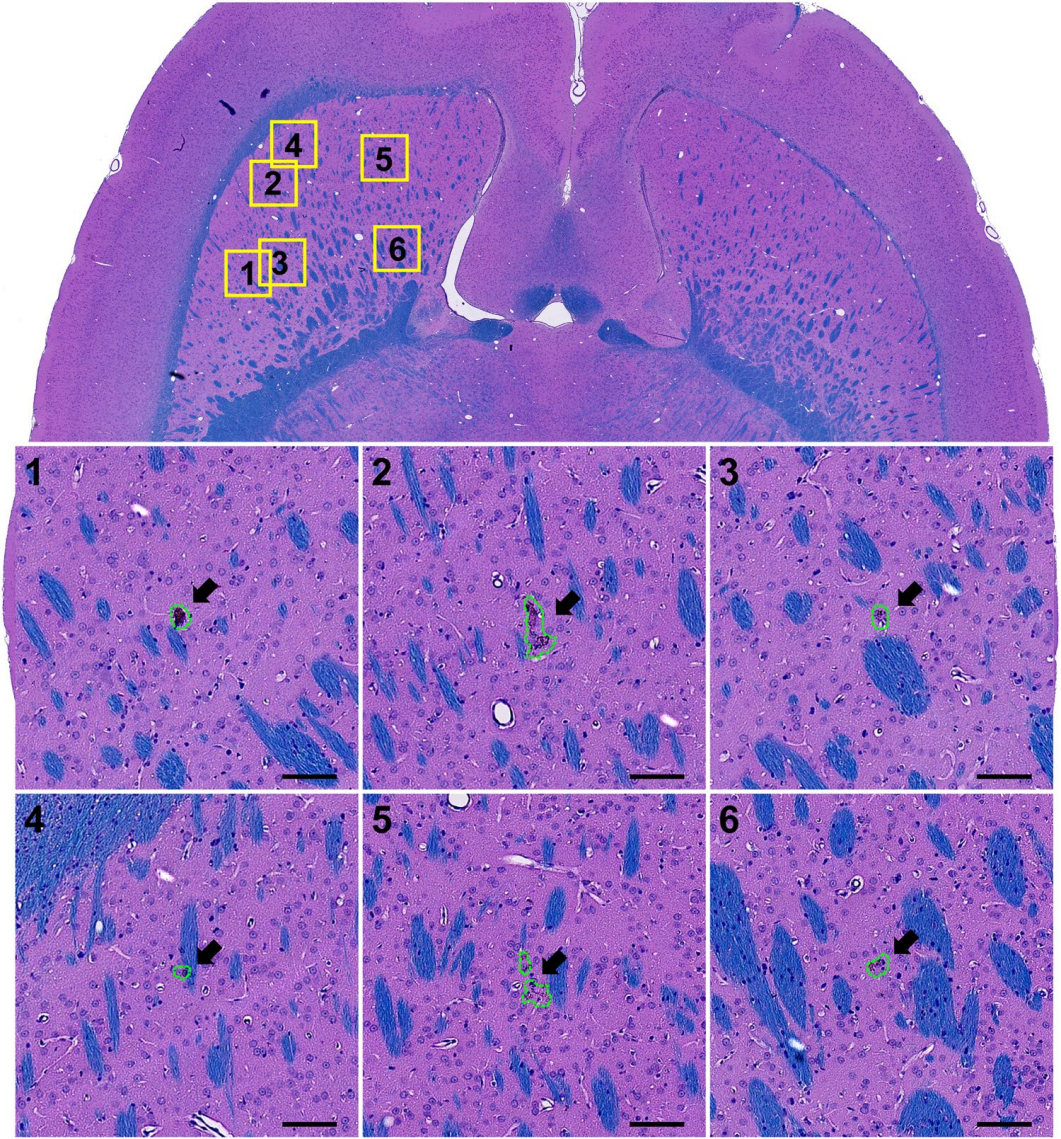
Microphotographs of a typical H&E-LFB stained section after three weekly sessions of FUS-induced BBB disruption and IN. Most of the brain appeared completely normal except for a few tiny clusters of hemosiderin particles. The segmentations used in measuring the dimensions and areas of these clusters are shown. Bars: 100 µm.

**Figure 10:**
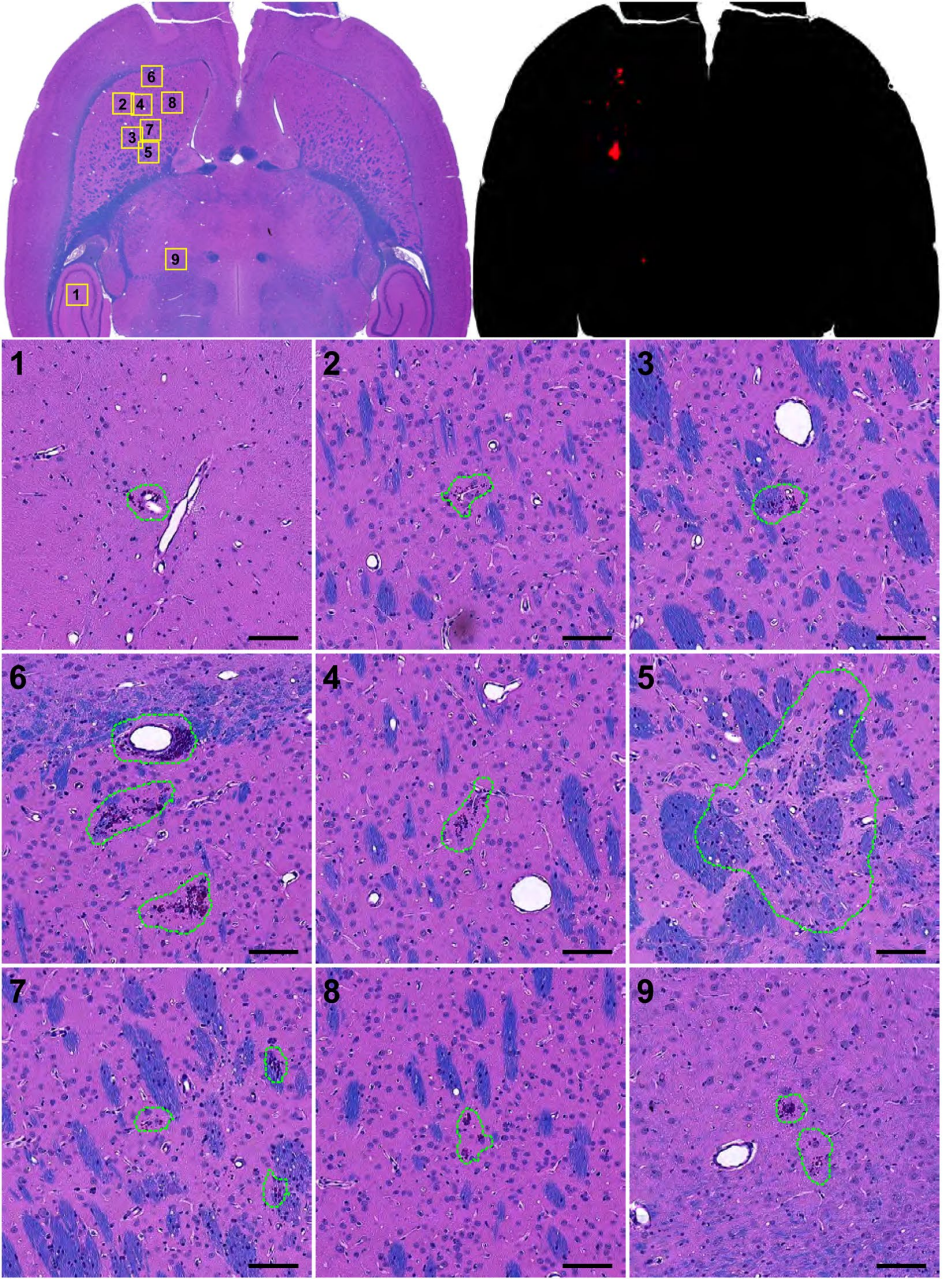
Microphotographs an H&E-LFB stained section after three weekly sessions of FUS-induced BBB disruption and irinotecan. This animal had the most severe effects: a few tiny petechiae and a small scar (region 5). Segmentation of all of the damage found is shown in the upper right plot. Bars: 100 µm.

A total of 28 H&E-LFB sections were investigated for FUS- and/or IN-induced neurotoxicity. The overwhelming majority of the sonicated regions appeared normal. A few clusters of extravasated erythrocytes (petechiae) or tiny hemosiderin particles, presumably indicative of vascular damage, were found in the sonicated hemisphere in 9/10 animals. The maximum dimension of these clusters was 59.7 ± 40.7 µm (range: 12.0–250 µm). Overall, 89% (111/126) of the clusters had dimensions less than 100 µm, and 14/15 of those that were larger occurred in Rat 2. One cluster of extravasated blood cells (dimensions: 228 × 88 µm, Rat 2), was found in the choroid plexus in the lateral ventricle. Figure 9 shows a typical example of the histological findings. Figure 10 shows a section obtained from Rat 2, where the largest petechiae were found. In four animals, a pale-stained area similar to that shown in (Fig. 8d,e) and Fig. 10 were observed. Three of these cases were those where hyperintense areas in the T2-weighted imaging did not resolve by 7 days after FUS; the fourth was the smallest of these pale-stained areas (dimensions: 0.5 × 0.25 mm).

### Experimental groups

Metrics and outcomes for the FUS-only and FUS + IN groups are listed in Table 2. Overall, the acoustic exposure levels, the presence and level of acoustic emissions, and the resulting tissue BBB disruption evident in by contrast-enhanced MRI were similar for the FUS-only and FUS + IN animals. Differences in peak acoustic power, administered acoustic energy, levels of harmonic emissions, and both R1 differences between hemispheres and signal enhancement in T1-weighted MRI after Gadavist injection were not significant (P > 0.05). More animals in the FUS + IN group had edema (9 vs. 6) and BBB disruption at 24 h (7 vs. 5) in location 1. The number of erythrocytes or hemosiderin clusters was similar for the two groups (47 vs. 43), but the dimensions and areas of the clusters were significantly (P < 0.001) larger in the FUS + IN group. Pale-stained areas (scars) were found in two of the 20 sonicated volumes in the FUS-only group; two more were found in FUS + IN group. No adverse health effects were detected among the animals who received FUS, IN, or their combination, and no significant difference was observed in the rate of weight increase between these groups.

**Table 2:** Measurements and outcomes for FUS-only, FUS + IN, and IN-only groups.

	All	FUS-only	FUS + IN	IN-only	P
Body weight increase (g/day):	1.8 ± 1.2	1.6 ± 1.0	1.2 ± 0.7	2.7 ± 1.5	0.12
Acoustic energy (mJ)	75.8 ± 16.1	74.9 ± 15.9	76.6 ± 16.2	—	0.08
Peak Harmonic emission (dB)	10.3 ± 2.7	10.2 ± 2.7	10.4 ± 2.8	—	0.19
Integrated Harmonic emission (dB)	282.1 ± 2.7	282.7 ± 2.7	281.6 ± 2.8	—	0.57
Reached controller goal (% locations)	98	97	98	—	—
Broadband emission (% locations)	2.7	2.1	3.3	—	—
Subharmonic emission (% locations)	4.2	3.4	5.0	—	—
ΔR1 (s^−1^)	0.075 ± 0.041	0.072 ± 0.036	0.078 ± 0.045	—	0.42
Contrast enhancement (% increase)	9.2 ± 5.7	9.7 ± 5.7	8.8 ± 5.7	——	0.40
Edema at 24 h (N volumes)^a^	15/116	6/60	9/56	—	—
Edema at 7d (N volumes)^b^	2/80	1/40	1/40	—	—
BBB disruption at 24 h (N volumes)^a^	12/116	5/60	7/56	—	—
Petechiae/hemosiderin: N volumes	12/40	8/20	4/20	—	—
Petechiae/hemosiderin: N clusters	91	48	43	—	—
Petechiae/hemosiderin: max. dimension (µm)	61.4 ± 46.6	38.6 ± 20.7	86.9 ± 53.9	—	<0.001
Petechiae/hemosiderin: area (µm^2^)	2709 ± 4552	929 ± 906	4697 ± 5984	—	<0.001
Scar: N volumes	4/40	2/20	2/20	—	—
Scar: max. dimension (mm)	0.72 ± 0.32	0.50, 0.69	0.51, 1.18	—	—
Scar: area (mm^2^)	0.17 ± 0.07	0.09, 0.25	0.12, 0.21	—	—

^a^No 24 h MRI in one animal.

^b^No 7d MRI after session 3.

There were significant (P < 0.05) differences in acoustic emissions and MRI-derived outcomes among the four sonicated volumes (Table 3). Volumes 1 and 2 had higher acoustic energy and lower harmonic emissions on average; volume 2 had more MRI contrast enhancement and greater changes in R1. However, the dimensions and areas of the petechiae/hemosiderin were significantly (P < 0.001) larger in volume 1, while volume 3 had more of these erythrocyte/hemosiderin clusters.

**Table 3:** Measurements and outcomes for the four sonicated volumes.

	Volume 1	Volume 2	Volume 3	Volume 4	P
Acoustic energy (mJ)	83.7 ± 14.4	78.9 ± 14.4	63.2 ± 10.7	68.5 ± 15.3	<0.001
Peak Harmonic emission (dB)	9.2 ± 2.5	9.5 ± 1.9	10.6 ± 2.1	11.6 ± 3.2	<0.001
Integrated Harmonic emission (dB)	276.4 ± 2.5	290.6 ± 1.9	292.8 ± 2.1	287.2 ± 3.2	<0.001
ΔR1 (s^−1^)	0.076 ± 0.040	0.101 ± 0.037	0.051 ± 0.035	0.062 ± 0.035	<0.001
Contrast enhancement (% increase)	7.3 ± 4.2	11.6 ± 5.2	7.5 ± 5.6	9.6 ± 6.4	0.001
Edema at 24 h (N sessions)^a^	15/29	0/29	0/29	0/29	—
Edema at 7d (N sessions)^b^	2/20	0/20	0/20	0/20	—
BBB disruption at 24 h (N sessions)^a^	12/29	0/29	0/29	0/29	—
Petechiae/hemosiderin: N volumes	4/10	2/10	4/10	2/10	—
Petechiae/hemosiderin: N clusters	32	3	48	8	—
Petechiae/hemosiderin: max. dimension (µm)	85.6 ± 61.2	48.4 ± 2.2	45.4 ± 28.9	66.2 ± 33.9	0.001
Petechiae/hemosiderin: area (µm^2^)	4992 ± 6867	1372 ± 76	1277 ± 1419	2672 ± 2313	0.003
Scar: N volumes	4/10	0/10	0/10	0/10	—
Scar: max. dimension (mm)	0.50-1.18	—	—	—	—
Scar: area (mm^2^)	0.09-0.25	—	—	—	—

^a^No 24 h MRI in one animal.

^b^No 7d MRI after session 3.

### Drug concentrations

Concentrations of IN and its active metabolite SN-38 were measured in sonicated and control brain tissue. In our first cohort, we injected IN 10 mg/kg and obtained brain tissue samples at two hours after sonication. The drug concentration was significantly higher (P < 0.01) in the sonicated hemisphere. However, SN-38 was below the lower limit of quantitation (~19 nM) in all 10 tissue samples from the sonicated and control regions. We sonicated a second cohort of animals treated with an IN dose of 200 mg/kg and examined drug concentrations at one hour after sonication. Again, the IN concentration was significantly higher (P < 0.001) with BBB disruption. SN-38 was measurable but near the lower limit of quantitation in 5/12 sonicated tissue samples (range, 18.0–23.9 nM), but measurable levels were not achieved in any of the control samples. Interestingly, the mean plasma concentration of SN-38 was similar for the two cohorts, despite the higher amount of drug administered and the earlier time the samples were acquired. In contrast, the plasma concentration of IN was 37 times higher in the second cohort. Concentrations are summarized in Table 4.

**Table 4:** Concentrations of irinotecan and SN-38 after BBB disruption.

Group	Irinotecan							SN-38		
	Plasma conc. (nM)	Tissue conc. (nM)				Tissue/plasma ratio		Plasma conc. (nM)	# detected	
		FUS	Control	Ratio	P	FUS	Control		FUS	Control
10 mg/kg at 2 h	3798	61.5	33.4	1.8	0.007	0.016	0.0089	645.9	0/10	0/10
	(16.6)	(61.1)	(36.9)	(59.4)		(53.6)	(28.4)	(20.0)		
200 mg/kg at 1 h	138500	5352.8	1154.6	4.6	0.000	0.041	0.0089	687.5	5/12^a^	0/12
	(41.4)	(70.6)	(31.7)	(71.4)		(49.7)	(41.0)	(20.1)		

^a^Concentration: 18.0–23.9 nM.

### Efficacy study

**Figure 11:**
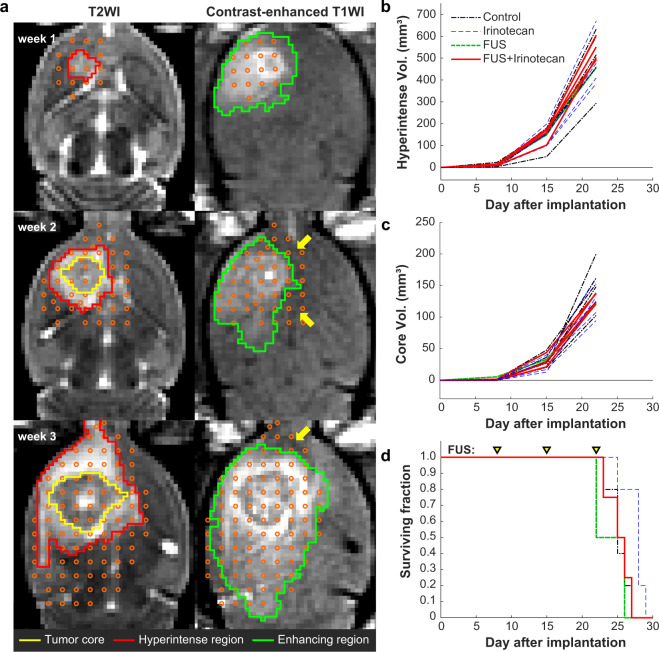
Efficacy study with BBB disruption and IN. **(a)** T2-weighted MRI (T2WI) and contrast-enhanced T1-weighted MRI (T1WI) of an F98 glioma over three weeks. Sonication targets were planned to cover the extent of the tumor core (yellow contours) as well as the hyperintense areas (red contours) evident in T2-weighted imaging. The plan included hyperintense areas evident in other planes. The resulting contrast enhanced area covered most of the planned region in most cases. Areas that were not enhancing in the central plane (arrows) were enhancing in other planes. (**b**,**c**) Tumor volume vs. time for animals that received drug only, BBB disruption only, drug and BBB disruption, or no treatment. No significant difference was observed among the groups in the volume of the hyperintense areas (**b**) or in the central core of the tumor (**c**). (**d**) Kaplan-Meier survival plots. No significant difference among treatment groups was observed.

We performed a pilot study testing whether weekly treatments with IV IN (20 mg/kg) and BBB disruption could reduce tumor growth or improve survival in the F98 glioma model. Results from this study are shown in Fig. 11. The number of targets required to cover the MRI-visible tumor and a surrounding margin increased from 18 in the first treatment, to 36–45 in the second, to 73–81 in the third (Fig. 11a, left). The contrast enhancement after the treatment included the tumor, although some targets had little or no enhancement (arrows); enhancement for those targets was observed in other imaging planes. While the animals tolerated the treatments, we did not observe a significant difference in tumor growth (Fig. 11b,c) or survival (Fig. 11d) in our first cohort of animals, and we decided not to continue. Additional findings from this study are shown in Supplemental Table 1.

## Discussion

These results demonstrate controlled, safe and repeated BBB disruption in a rat model using a low-frequency clinical TcMRgFUS system. Disruption was achieved in nearly every target in the sonicated volumes over 30 consecutive sessions. Closed-loop feedback control was successful in tailoring the acoustic power level to a pre-determined level of cavitation activity while minimizing wideband emission, a signature for inertial cavitation^22^. This is a promising finding, since tumor and other disease models are uncommon in larger animals. Being able to perform this procedure in rats is also substantially less expensive and more practical than in larger animals.

There was no significant difference in the acoustic energy applied, the acoustic emissions recorded during sonication, or in the resulting BBB disruption between the FUS + IN and the FUS-Only animals. No adverse effects of the drugs on the animals’ health were noticed, and no signifcant difference in the the rate of weight gain was observed between the two groups. We did find that the size of the erythrocyte/hemosiderin clusters in the H&E-LFB stained sections was larger in the FUS + IN group. These clusters are indicative vascular damage and petechiae. This finding could suggest that IN exasperated the vascular damage or perhaps impeded its repair. It is consistent with a previous study that found that delivery of doxorubicin across the BBB resulted in more damage when MRI-evident petechiae were observed^23^. It would be interesting to explore this effect further in studies with higher exposure levels and more vascular damage.

The histological effects were conistent with numerous prior preclinical studies on FUS-induced BBB disruption using different acoustic frequencies and transducer geometries^24–26^. Overall, the brains tissue appeared normal in standard H&E-LFB stained sections, with some evidence of minor vascular damage. In some cases, extravasated erythrocytes were evident in the brain parenchyma or surrounding blood vessels. Such cases likely occurred during session 3, which was 48 h before the animals were euthanized. Hemosiderin deposits are the result of phaogcytosis of red blood cells and hemoglobin by macrophages, presumably after mild vascular damage that occurred during the first two sessions. The largest example of extravasated blood cells occurred in the animal shown in Fig. 10, where a few small accummulations of erythrocytes was observed around a relatively large blood vessel. Other than in this animal, only 1 hemosiderin cluster with dimensions greater than 100 µm was found.

In four animals, a small pale-stained region was found in the putamen in sonication volume 1. These areas appeared to be scars and are consistent with histological effects observed after ischemic tissue damage. Such damage has been observed to a larger degree in earlier work that evaluated the safety of multiple sessions of BBB disruption without closed-loop control^26^. Those studies, which disrupted the BBB in relatively small areas, produced larger regions of tissue damage than was observed here where the barrier was disrupted across an entire hemisphere. While such damage is not desireable, given their small size (1.2 mm or less) and low occurrence, such damage would likely be an acceptible risk for brain tumor patients who currently have no effective treatment options. Other applications may have different requirements for safety.

These small lesions could suggest that our sonication overlap was too aggressive in the striatum. The striatum was the largest contiguous structure targeted here, and in in the center of the sonication pattern the effects of many sonications may have accumulated to a degree not present in other tissue structures. The acoustic energy density was substantially higher in the striatum (Fig. 1c), and this appeared to be reflected in several cases in the resulting BBB disruption (Fig. 10). Others have found that excessive sonication durations^27^ and large microbubble doses^28^, both of which can increase the “magnitude” of the BBB disruption, can result in brain tissue damage. Perhaps it is possible to have too much BBB disruption. Indeed, our subsequent work using a less dense sonication pattern did not observe the tiny damage we found here^20^.

These results suggest that the passive cavitation detector we used to monitor broadband emissions may not sensitive enough to detect inertial cavitation of a small number of microbubbles. Our post-FUS imaging also did not detect the vascular damage; we did not see clear evidence of hypointensity in T2*-weighted imaging. Both may not be surprising given the small size of petechiae/hemosiderin clusters (mean size: 59.7 ± 40.7 µm) and their sparsity. It is likely that in many cases, a tiny number of microbubbles were responsible for the observed tissue effects. To fully avoid these effects, more efforts are needed to improve the sensitivity of passive cavitation detectors. These efforts will be more important when monitoring acoustic emissions through the human skull, which will further attenuate them.

Strong increases in harmonic emissions were observed in 98% of the 1071 targets sonicated in these rats where passive cavitation recordings were made, and the closed-loop controller was successful in modulating the power at each individual target to ensure a repeatable level of emission. Increases in harmonic emissions after administration of microbubbles thus appear to be a useful metric to ensure that the exposure level is sufficient to disrupt the BBB. These results are consistent with several previous studies using small animal FUS systems^29–32^, and the controller used here is similar to one described recently by Sun *et al*.^33^.

Despite having a consistent level of harmonic emissions, the amount of MRI contrast agent delivered to the brain varied significantly. Previous work in rodents and macaques showed a good correlation between the harmonic emission recordings and the level of BBB disruption measured using MRI methods^29–32^. It is not clear why that was not the case here. It could be simply that the signal-to-noise ratio of our recordings were not as strong as those earlier studies, and that was reflected by a more variable level of BBB disruption. Our detector was placed near the face of the array, nearly 15 cm away from the focal region. The pressure amplitude of the acoustic emissions decreases as the square of the distance and it may be beneficial to move the detector closer to the focus. Using multiple detectors could also decrease the variability.

There was also considerable variabilty in the BBB disruption among the four volumetric sonications. We suspect this was due to cumulative effects of the overlapping sonications, perhaps reflected by the variations in total acoustic energy density delivered to the brain (Fig. 1c). Different vascular densities in different tissue structures may have also contributed to this variability. Better control might be possible with an infusion of microbubbles instead of using bolus injections^33^ since the microbubble concentration would be stable over a longer time. Having this longer time might allow for more consistent exposures overall. However, to avoid excessive microbubble doses, the peak concentration may be lower with an infusion, which could pose detection challenges through a human skull.

We were not able to utilize the control approach described by O’Reilly *et al*., where the exposure level is increased until subharmonic emissions are observed, at which time the pressure amlitude is dropped by a specified ratio^34^. The onset of subharmonic emission is thought to occur at a specific focal pressure amplitude, so this approach is promising in calibrating the exposure level at the focus. We attempted to use this approach in a pilot study (data not shown), but we were unable to achieve BBB disruption without substantial damage. We suspect that the subharmonic detectors were perhaps not sensitive enough to detect the small subharmonic signal, or that the step size in acoustic power that we used was too high.

One animal in the FUS + IN group died after the third treatment while obtaining post-FUS imaging. We believe that this was due to hypothermia caused by neglecting to cover the animal with a blanket after the sonications, not to any unexpected neurotoxicity. Other than finding dark neurons in both hemispheres (indicative of ischemia or an artifact from inadequate perfusion fixation^35^), there was nothing abnormal in the histology that would suggest that the sonications or the IN could be the cause of death.

### Drug delivery and efficacy

We also evaluated the safety of repeatedly delivering IN to the healthy brain. Irinotecan is a topoisomerase inhibitor used to treat metastatic colon and rectal cancers. Several studies have evaluated IN in combination with other agents in patients with malignant glioma^36,37^. When used with bevacizumab, six-month survival improved to 46% compared to 21% in patients who received temozolomide^38^. Recurrence occurs in almost all patients, even when complete resection of the tumor mass is achieved. Malignant gliomas are highly infiltrative, and if one could safely and repeatedly administer an effective chemotherapy agent to the surrounding healthy brain, one could potentially reduce recurrence. These results demonstrate that such delivery is possible in rats without inducing significant neurotoxicity.

We evaluated the concentrations of IN and its active metabolite SN-38 in the brain after FUS-induced BBB disruption. While we saw significant enhancement of IN, SN-38 only achieved measurable levels in animals treated with a very high IN dose, but not in all animals. IN is metabolically converted to SN-38 by carboxyesterases predominantly in the liver, and it is possible that the BBB was largely or completely restored before peak plasma concentrations of SN-38 were achieved. However, based upon the observed plasma concentrations of SN-38, the estimated concentration of SN-38 in the brain would be on the order of 10 nM if the tissue/plasma concentration ratio was similar to IN, which could not be measured by the analytical method used in this study. Further, others have suggested that SN-38 might be metabolized by glioma cells^39^. Thus, despite the low SN-38 concentrations in the brain, we conducted an efficacy study to see if enhanced IN delivery and perhaps intratumoral conversion of IN to SN-38 could slow tumor growth and improve survival. We did not see these effects, and thus moved on to a different chemotherapy agent^20^. Given the clinical promise of IN for glioma patients, it might be useful to further study this drug in the context of BBB disruption by exploring alternative sonication/drug administration schedules or evaluating SN-38 concentrations in tumors using analytical methods with greater sensitivity. Previous results suggest a small survival benefit in the F98 model in rats can be achieved with IN alone^40^. However, *in vitro* studies also suggest that high concentrations of IN^41^ and effective conversion to SN-38^40^ are needed to produce toxicity in this cell line. Perhaps a different tumor cell line that is more representative of the human glioma sensitivity of this drug would provide different results.

## Conclusions

This work shows that ExAblate Neuro low-frequency clinical TcMRgFUS system can be used to repeatedly and reliably disrupt the BBB in a rat model. An integrated closed-loop control system based on recordings of the increase in acoustic emissions produced by the presence of Definity microbubbles ensured that BBB disruption occurred without clinically significant vascular damage. The delivery of IN to the brain was not neurotoxic, however it resulted in low concentrations of the active metabolite SN-38 in the brain and was not effective in reducing tumor growth or prolonging survival in F98 glioma.

## Methods

### Animals

All experiments were approved by the Institutional Animal Care and Use Committee at Brigham and Women’s Hospital. The animals were housed, fed, and watered according to the Office of Laboratory Animal Welfare and the Association for Assessment and Accreditation of Laboratory Care regulations. The experiments were performed using 15 Sprague-Dawley rats (8 males, 7 females) in the safety study, 16 male Sprague-Dawley rats in the study measuring drug concentrations, and 16 Fischer rats (7 males, 9 females) in the tumor studies. They were anesthetized with ketamine (80 ml/kg) and xylazine (10 ml/kg) administered as needed IP. The fur on the scalp was removed with clippers and depilatory cream, and the tail vein was catheterized. An acrylic stereotactic frame was constructed that allowed for repeatable placement on the TcMRgFUS system with only the top of head submerged in water. The animals were placed supine on the device and kept warm using a heated water blanket.

### Safety study

We first evaluated the safety of repeatedly disrupting the BBB with the TcMRgFUS device. The animals were randomly divided into three groups (N = 5 for each). The first group (FUS-only) received three weekly sessions with FUS-induced BBB disruption. The second (FUS + IN) received three sessions with FUS-induced BBB disruption and IN. The third (IN-only) received three sessions of IN. Animals in the first two groups underwent MRI immediately after FUS and at 24 hours. We did not obtain MRI for the IN-only animals. Due to drug availability, the third session in two FUS + IN animals was delayed by one week. The animals’ weight was recorded regularly, and any adverse effects on the animals’ appearance or behavior were noted.

Two days after the last session, the animals were deeply anesthetized and euthanized via transcardial perfusion with formalin. The brain was then removed and immersed in formalin. It was cut into three axial blocks and photographed. Using these pictures and the MRI as a guide, selected blocks were paraffinized, cut in 5 µm sections, and stained with H&E and Luxol-Fast Blue. One animal in the FUS + IN group was found dead after post-FUS MRI. Presumably, this was the result of neglecting to cover the animal with a blanket after anesthesia. Two animals in the IN-only group also died while recovering from anesthesia under a heat lamp after the second session. These two animals were excluded from the study, and two animals were added that received the full three sessions.

### Irinotecan administration

In the safety study, Irinotecan hydrochloride (Areva Pharmaceuticals, Georgetown, Indiana, USA) was injected at a dose of 10 mg/kg intravenously over a period of approximately 10 s. Immediately prior to chemotherapy administration, atropine was administered at a dose of 0.01 mg/kg to reduce the cholinergic reaction to IN. In the FUS + IN animals, the drug was administered after the first two volumetric sonications to investigate whether the order of BBB disruption and drug administration influenced the tissue effects. The order of the sonications was reversed in half the animals. Due to low concentrations of IN and its metabolite SN-38 measured in the brain, we increased the dose to 20 mg/kg in the efficacy study.

### Tumor studies

Wild-type F98 cells (passage number six, provided by Rolf F. Barth^42^ at the Department of Pathology, The Ohio State University, Columbus, OH, USA) were cultured in Dulbecco’s modified Eagle medium (1×) supplemented with 10% FBS and 0.1% Penicillin Streptomycin in a humidified incubator with 5% CO_2_ at 37 °C. Following the surgical procedure as previously described^33^, a 4-μL cell suspension (2 × 10^4^ cells) was injected into the right caudate putamen 3.5 mm from the dura surface using a 10-μL gastight Hamilton syringe in Fischer rats. Animal behavior was monitored daily after surgery and the sutures were removed 5 days later.

The rats were divided randomly into three groups (control, N = 5; drug-only, N = 5; FUS-only: N = 2; FUS + IN, N = 4). On day 8 after implantation they were imaged to confirm tumor growth. FUS + IN rats received microbubble-enhanced FUS followed by IN administration. Drug-only rats received drug at this time. Treatments were repeated at one and two weeks later. The animals were imaged weekly to monitor tumor growth. Animals were euthanized when they exhibited severely impaired activity, weight loss exceeding 20% within one week, or tumor core dimensions exceeding 10–11 mm.

### Equipment

The clinical TcMRgFUS system tested was ExAblate Neuro low-frequency device (InSightec, Haifa, Israel). This system uses a 1024-element, 230 kHz hemispherical transducer with a diameter of 30 cm with an integrated cavitation monitoring system, and a water cooling/degassing/circulating system^3^. The transducer was placed on its side so that it could be filled with water like a bowl for use in animals (Fig. 1a). Electronic beam steering with the phased array transducer enabled rapid targeting to multiple sites during each sonication. The center depth of the brain was approximately 1 cm above the geometric focal plane of the TcMRgFUS transducer. No aberration correction was used to compensate for the rat skull.

The TcMRgFUS system was integrated into a 3 T clinical MRI machine (Signa HDxt, GE Healthcare, Milwaukee, WI, USA). A rectangular receive-only surface coil (dimensions: 5 × 6 cm) was constructed for use in rat experiments. It was mounted so that it was partially submerged below the water level of the TcMRgFUS device (Fig. 1a). We drained the water from the transducer for MRI to avoid artifacts and improve SNR.

Axial 3D T2*-weighted spoiled gradient echo images (TR/TE: 33.3/19.0; flip angle: 15°; FOV: 8 × 8 × 2.1 cm; matrix: 256 × 256 × 30; bandwidth: ±15.6 kHz; averages: (1) were used to select the sonication targets and to detect petechiae after sonication. Axial T1-weighted fast spin echo (FSE) imaging (TR/TE: 500/13.8 ms; ETL: 4; FOV: 8 × 8 cm; slice thickness: 1.5 mm; matrix: 256 × 256 bandwidth: ±15.6 kHz; averages: (4) was obtained. This imaging was repeated five times (four axial, 1 sagittal acquisition) after IV injection of 0.125 mmol/kg mmol Gadavist (Bayer HealthCare Pharmaceuticals, Inc., Whippany, Hanover, NJ, USA). Finally, we estimated Gadavist concentrations by measuring R1 relaxation. For this, we used a FSE sequence repeated with multiple TR’s (TR: 6000/3200/1600/800/400/200/100 ms; TE: 13.4 ms; ETL: 3; FOV: 9 × 9 cm; slice thickness: 2 mm; matrix: 256 × 256; bandwidth: 15.6 kHz; averages: 1 for TR = 6000/3200/1600 ms, 2 for TR = 800/400 ms, 4 for TR = 200/100 ms). R1 relaxation was related to Gadavist concentrations using the relaxivity of Gadavist – 4.44 s^−1^·mmol^−1^ ^21^.

The T2*- and contrast-enhanced T1-weighted MRI were repeated 24 h after the sonications. In addition, T2-weighted FSE images (TR/TE: 4000/81.7 ms; ETL: 12; FOV: 9 × 9 cm; slice thickness: 2 mm; matrix: 256 × 256 bandwidth: ±15.6 kHz; averages: (1) were obtained at that time to evaluate whether any edema was present. In the second and third FUS sessions, T2-weighted imaging was also acquired before the sonications to evaluate whether edema observed at 24 h was resolved. In the tumor studies, we monitored tumor growth with weekly T2-weighted imaging.

### Sonications

In healthy rats, 36 overlapping targets were sonicated with an aim of disrupting the BBB in a volume that covered most of the cerebrum in the right hemisphere at a central axial plane. These targets were selected with a 1 mm center-to-center spacing and arranged to conform to the shape of the rat brain (Fig. 1b). Direct sonication on the ventricles was minimized. With this overlap, simulations of the acoustic field of the 36 locations suggest that the highest total acoustic energy density was in the striatum (Fig. 1c). These target locations were originally planned on 3D T2*-weighted MRI of a rat that was obtained earlier. Before the sonications in each rat, the pre-FUS T2*-weighted MRI were manually registered to this template using software developed in-house. Each volumetric sonication targeted 9 locations (Fig. 1b); four sonications were delivered to each rat. The four sonications were first delivered without microbubbles (total duration: 30 s), and then they were repeated with microbubbles (total duration: 55 s). For BBB disruption, Definity microbubbles (Lantheus Medical Imaging, North Billerica, MA, USA) were administered at the start of the 55 s sonication as a bolus injection at a dose of 10 µl/kg. To facilitate injecting such a small volume, the agent was diluted in PBS at a ratio of 10:1 followed by a 200 µl injection of saline. The sonications consisted of 5 ms bursts. The bursts were applied sequentially to the nine targets at an interval of 101.6 ms; the pulse repetition frequency for each target was thus 1.1 Hz. A delay of at least two minutes between sonications ensured that most of the microbubbles had cleared at the start of each sonication. The entire procedure, from the first to the last MRI was approximately 80 min. In the tumor study, we used 1 mm spacing and placed the targets to cover the tumor core and the hyperintense region evident on T2-weighted MRI.

Sonication began at an acoustic power level of 0.16 W and was not allowed to exceed 0.39 W. Based on calibrations provided by the manufacturer, these levels corresponded to peak negative pressures of 68 and 165 kPa, respectively, in water. The insertion loss due to the skull bone in the rat is expected to be approximately 5% at this frequency^43^. The power was actively controlled based on acoustic emissions recordings obtained with two hydrophones. One had resonant frequency of 115 kHz and was integrated into the TcMRgFUS device. Filtration and amplification of this hydrophone was provided by the device manufacturer. The second hydrophone was constructed in house and was a 5 × 3 cm elliptical transducer with a resonant frequency of approximately 660 kHz. It was mounted at the bottom of the TcMRgFUS transducer (Fig. 1a). Filtration (band-reject, Butterworth, 0.133–0.391 MHz) and 20 dB amplification was provided by a programmable filter (model 3944, Krohn-Hite, Brockton, MA, USA). The emissions were recorded using the TcMRgFUS device. With each 5 ms burst, 4.1 ms of data were obtained at a sample rate of 2 × 10^6^ samples per second. The TcMRgFUS system provided the spectra obtained with each burst in real-time to a PC for analysis to control the power using software developed in-house in Matlab. Communication between this PC and the TcMRgFUS device was supplied by the manufacturer.

The allowed power levels and the other controller settings were established in pilot studies (data not shown) and aimed to be conservative while minimizing false detection. Spectra were obtained for 3.5 s before each sonication to determine the noise floor. The microbubbles were injected at the start of the sonication; the controller 8 s later. A proportional controller that used the mean of the signal strength at the second and third harmonics modulated the power until a pre-determined level of harmonic emissions (between 6-7.5 dB above the noise floor) was achieved. If the level of harmonic emissions (*H)* for a burst was outside of this range, the power for the next burst was set based on the following equation:

$$Powe{r}_{new}={(\sqrt{Powe{r}_{current}}-{P}_{gain}\times (H-|{H}_{goals}|))}^{2}$$

where the constant *P*_*gain*_ was 0.0167. This controller assumed that the strength of the harmonic enhancement in dB was proportional to the pressure amplitude^33^.

Since after injection the microbubble concentration decreases over time, the power was only modulated between 8 and 25 s. After this time, the power was fixed to the mean value of all bursts where *H*_*goal*_ was achieved. If at any time subharmonic emission (115 ± 10 kHz) was detected by the first hydrophone or broadband emission (660 ± 40 kHz) by the second, the power level was reduced by 40%, and the power level was fixed for the remainder of the sonication. Harmonic emissions were obtained at the second and third harmonic of the TcMRgFUS device (460 and 690 ± 10 kHz).

The emissions were calculated in dB relative to the noise floor, which was obtained before each sonication. During the first 8 s of each sonication (before the microbubbles arrived in the brain), a second noise floor measurement was obtained during bursts applied at 0.16 W, which presumably had no broadband emissions. The threshold for subharmonic emissions was defined to be 3.3 standard deviations above this noise floor; it was set to 4 standard deviations above this noise floor for broadband emissions. Acoustic emissions recordings from four volumetric sonications without microbubbles and one with microbubbles were excluded due to a malfunctioning cable.

### Irinotecan plasma and tissue concentrations

We measured concentrations of IN and its active metabolite SN-38 in plasma by high performance liquid chromatography with fluorescence detection as previously described with minor modifications^44^. The brains were extracted and biopsies (diameter: three mm; approximately 50 mg) were obtained. Frozen tissue samples were thawed, rinsed three times with ice-cold phosphate buffered saline, gently blotting with filter paper between each rinsing, and weighed in a microcentrifuge tube (range, 15.7–26.2 mg). After adding 300 µL of ice-cold water, the tissue was homogenized for 4-min using an Ultra-Turrax T8 disperser with an S8N-5G dispersing element (IKA Works, Inc., Wilmington, NC, USA). The homogenate was sonicated for 5-min, subjected to three freeze-thaw cycles, and centrifuged (12,000 *g*, 10-min). Tissue homogenates were assayed in the same manner as plasma samples. The lower limits of quantitation were 5.0 ng/mL (8.5 nM) and 0.50 ng/mL (1.27 nM) for the determination of IN and SN-38, respectively, in plasma. The effective lower limit of quantitation for measuring both analytes in tissue samples was effectively 15-times higher than plasma because of dilution to prepare the homogenate. Samples from two cohorts of animals were analyzed. In the first, we administered IN at a dose of 10 mg/kg and euthanized the animal for tissue extraction two hours later. In the second, 200 mg/kg IN was administered, and the tissue samples were obtained one hour later. We compared drug concentrations from location 1 in Fig. 1b and the corresponding nonsonicated region in the left hemisphere in the first cohort; concentrations from locations 1 and 3 were analyzed in the second cohort.

### Data analysis

All analysis was performed in Matlab (Mathworks, Natick, MA, USA). Maps of the R1 relaxation rate were created using a method described elsewhere^45^. The “nlinfit” command in Matlab was used for the nonlinear regression. Three axial images were obtained. Different tissue structures were manually segmented in the center slice by one user (NM). Regions including a diameter of 2.5 mm centered on each of the 36 targets, along with corresponding targets in the contralateral hemisphere, were considered. Contrast enhancement in the T1-weighted FSE images was calculated as a percent increase relative to imaging obtained before Gadavist injection. It was estimated in five planes, and a maximum intensity projection was performed after segmenting the brain. The mean signal enhancement in these projections was measured for the areas covered by the four volumetric sonications and corresponding locations in the contralateral hemisphere. Statistical comparisons of the acoustic emissions and MRI-based measurements between the sonicated and non-sonicated control hemispheres and among the four sonicated regions were made using one-way ANOVA; P < 0.05 was considered significant. H&E-LFB stained sections were scanned at 20× resolution. They were evaluated by one author (NV) without knowledge of experimental group or which hemisphere was sonicated. All abnormalities were segmented, and their dimensions and area calculated. IN tissue concentrations are reported as the geometric mean (geometric %CV). Comparisons between sonicated and control tissue samples used a paired two-tailed t-test of log transformed data. In the tumor study, the tumor core and the hyperintense regions were manually segmented to estimate tumor volume; volumes among different treatment groups were compared using one-way ANOVA. Survival was compared using Kaplan-Meier analysis and the log-rank test.

## Supplementary information

Supplementary information.
