# Supplementary material for: Blood-brain barrier disruption and delivery of irinotecan in a rat model using a clinical transcranial MRI-guided focused ultrasound system
Source: Sci Rep. 2020 May 29;10:8766. doi: 10.1038/s41598-020-65617-6 (PMC7260193; doi:10.1038/s41598-020-65617-6)
Supplement: Supplementary file 1 — Supplementary information. [file 41598_2020_65617_MOESM1_ESM.docx]

Blood-brain barrier disruption and delivery of irinotecan in a rat model using a clinical transcranial MRI-guided focused ultrasound system

Nathan McDannold*, PhD¹, Yongzhi Zhang, MD¹, Jeffrey G. Supko, PhD², Chanikarn Power, BS¹, Tao Sun, PhD¹, Natalia Vykhodtseva, PhD¹, Alexandra J. Golby, MD¹^,^ ³, David A. Reardon, MD^4^

¹ Department of Radiology, Brigham and Women’s Hospital, Harvard Medical School, Boston, MA

² Department of Medicine, Massachusetts General Hospital, Harvard Medical School, Boston, MA

³ Department of Neurosurgery, Brigham and Women’s Hospital, Harvard Medical School, Boston, MA

^4^ Department of Medical Oncology, Dana-Farber Cancer Institute; Department of Medicine, Brigham and Women’s Hospital; Harvard Medical School, Boston, MA


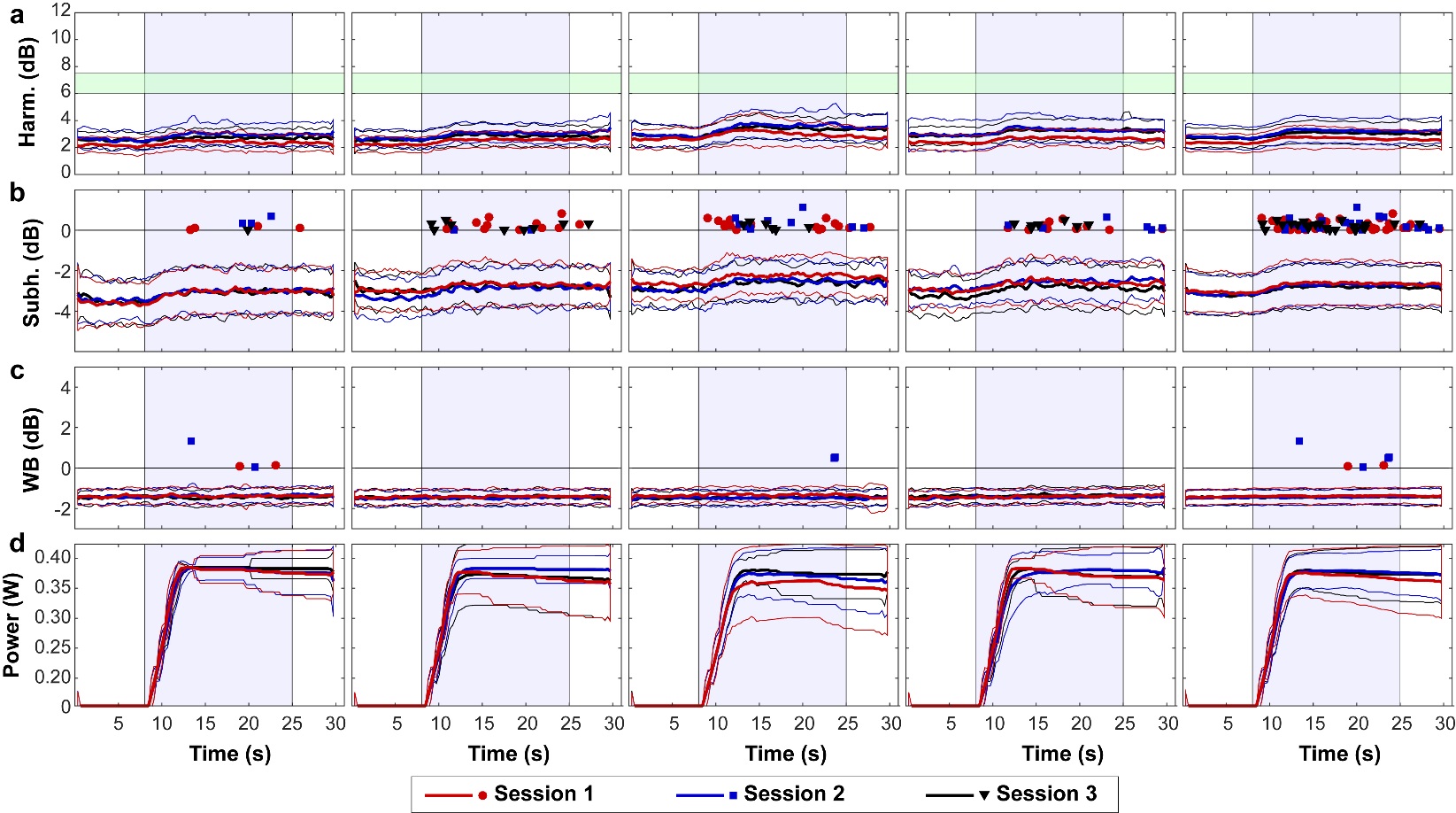


**Supplemental Figure 1:** Mean acoustic emissions and acoustic power vs. time for all animals for the four volumetric sonications without microbubbles. These sonications were performed immediately before the sonications with microbubbles. A. Harmonic emissions were the basis to control the power level at each target during sonication; the controller allowed the power to increase between 8-25 s. Without microbubbles, they rarely reached the controller goals (green box) B. Subharmonic emissions, plotted here in dB relative to the threshold used to trigger a reduction in acoustic power, were rarely detected. The triggers that occurred are noted by the filled symbols and generally were only occurred at an amplitude slightly above this threshold. C. Broadband emission, again plotted here in dB relative to the threshold that triggered a reduction in power, rarely occurred. D. The acoustic power varied much less than during the microbubble-enhanced sonications shown in **Figure 3.** (Thick lines: mean values; thin lines/shaded areas: ± 1 standard deviation).

**Supplemental Table 1:** Results from Efficacy study

HIV: Hyperintense volume

*Estimated time required for tumor to double in volume; based on exponential fit of data in **Figure 11**
